# Hepatic epithelioid hemangioendothelioma—a single-institution experience with 51 cases

**DOI:** 10.3389/fonc.2023.1236134

**Published:** 2023-08-03

**Authors:** Lei Feng, Manjie Li, Zhuo Huang, Mingqing Xu

**Affiliations:** ^1^ Division of Biliary Surgery, Department of General Surgery, West China Hospital, Sichuan University, Chengdu, Sichuan, China; ^2^ Radiology Department of West China Tianfu Hospital, Sichuan University, Chengdu, Sichuan, China; ^3^ Department of Pathology, West China Hospital, Sichuan University, Chengdu, Sichuan, China; ^4^ Division of Liver Surgery, Department of General Surgery, West China Hospital, Sichuan University, Chengdu, Sichuan, China

**Keywords:** hepatic epithelioid hemangioendothelioma, clinicopathologic features, therapeutic strategies, follow-up, prognosis

## Abstract

**Objectives:**

The aim of the present study was to describe the experience at a single institution in the management of hepatic epithelioid hemangioendothelioma (HEHE).

**Methods:**

We included 51 patients with histologically confirmed HEHE. We performed log-rank (Cox–Mantel) survival analyses using Kaplan–Meier methods to test differences in survival between patients in different groups. Univariate Cox regression analyses and multivariate proportional hazards regression model were carried out to identify independent prognostic factors.

**Results:**

Different imaging modalities were used to diagnose HEHE with various presentations. Liver resection (LR), liver transplantation (LT), systemic treatment (ST), and surveillance had been used in our study. A significant difference was noted between the LR group and the surveillance group with respect to mean survival (*p* = 0.006), as was in the LR group and the ST group (*p* = 0.036), and in surgical approach (LR and LT) and nonsurgical approach (ST and surveillance) (*p* = 0.008). The mean survival between the ST group and the surveillance group was not significantly different (*p* = 0.851). LR (p = 0.010) and surgical approach (p = 0.014) were favorable predictors of outcome, while macrovascular invasion (MaVI) (p = 0.037), lung metastasis (p = 0.040), and surveillance (p = 0.033) were poor prognostic factors in univariate analysis. Multivariate analysis showed that LR (p = 0.010) and surgical approach (p = 0.014) were independently associated with good OS, while surveillance (p = 0.033) was independently associated with poor OS. After adjusting for confounding factors, patients in the LR group have much better OS than those in the surveillance group (p = 0.013). However, there was no significant difference in OS between the LR group and ST group (p = 0.254), as was in the ST group and the surveillance group (p = 0.857).

**Conclusions:**

The definitive diagnosis of HEHE was dependent on histopathology, and it was not possible to make a specific diagnosis without biopsy because the radiological findings were similar to those in some hepatic malignancies. ST was not recommended for patients who were not candidates for surgical approaches, and surgical approaches should be warranted regardless of disease stage. The retrospective nature and the small size of the data limited the generalizability of the study, designing a worldwide database that contains all data about patients with HEHE independent of their therapy, which was highly recommended.

## Introduction

Hepatic epithelioid hemangioendothelioma (HEHE) is a low-to-moderate malignant tumor (1) ranging in malignancy between hepatic hemangioma (HHA) and hepatic hemangiosarcoma (HAS) (2), with a reported incidence of 1–2 for every 1 million people (3). Clinically, HEHE presents as an incidental finding at the time of diagnosis with symptoms ranging from nonspecific to liver failure at advanced stage (4). The characteristic clinical manifestations and mechanisms underlying the development of HEHE are still unclear.

The distribution pattern of HEHE can be characterized as uninodular, multinodular, or diffuse disease, with the multifocal form being the most common. An appearance of diffuse disease can be observed in the late stage of focal lesions, associated with infiltration of hepatic vein (HV) and portal vein (PV), and often accompanied by distant metastasis. The lung, peritoneum, spleen, lymph nodes, and bone are the most common sites of extrahepatic metastasis (5).

Most patients are initially misdiagnosed due to variable and atypical clinical manifestations (6). The imaging modalities used in the diagnosis of HEHE are varied, such as contrast-enhanced computed tomography (CT), contrast-enhanced magnetic resonance imaging (MRI), conventional ultrasonography (CUS), and contrast-enhanced ultrasonography (CEUS). For some patients, gadolinium ethoxybenzyl diethylenetriamine pentaacetic acid (Gd-EOB-DTPA)-enhanced MRI (Gd-EOB-DTPA-enhanced MRI) and 2-[^18^F] fluoro-2-deoxy-D-glucose (^18^F-FDG) positron emission tomography/computed tomography (PET/CT) (^18^F−FDG PET/CT) were further performed. The suggestive imaging features of HEHE are the peripheral location of the nodules, the hepatic capsular retraction, the lollipop sign, the target sign, and the tendency of multiple lesions to coalesce (7, 8). However, the imaging features of HEHE are nonspecific, and differentiation from metastatic carcinoma (MCA), multifocal liver cancer, intrahepatic cholangiocarcinoma (ICC), HHA, HAS, and other conditions must be achieved. Pathological findings play a decisive role in the diagnosis of HEHE, with immunohistochemical (IHC) detection of endothelial and vascular endothelial markers such as cluster of differentiation (CD) 31 (CD31), CD34, ERG, friend leukemia integration 1 transcription factor (Fli-1), and factor VIII-antigen (FVIII) as the basis of diagnosis (3).

There are no consensus treatment protocols, and strategies including liver resection (LR), liver transplantation (LT), systemic treatment (ST), and surveillance have all been used with varying outcomes.

The aim of the present study was to describe the experience at the largest-volume liver cancer and transplantation center in southwest China using multiple approaches in the diagnosis and management of HEHE.

## Materials and methods

### Study population

This is a single-institution retrospective observational study. We included 51 patients with histologically confirmed HEHE between 1 January 2011 and 31 December 2021 at West China Hospital of Sichuan University. All procedures performed in this study involving human participants were in accordance with the Declaration of Helsinki (as revised in 2013). The retrospective study was approved by the Ethics Committee of West China Hospital of Sichuan University and waived the need for informed consent (2022 Year of Approval No. 645) and has been registered with the Chinese Clinical Trial Registry under the registration number ChiCTR2200060969.

The treatment options for patients with HEHE include LR, LT, ST, and even surveillance without any therapy. Treatment strategies were individualized based on disease stage, liver function, and patient’s performance status (PS). LR was considered for patients with Child–Pugh A/B grade of liver function, PS score ≤ 2, number of lesions ≤ 3, and without imaging evidence of extrahepatic metastasis. If the lesion was confined to the same segment or the same half of the liver, even if the number of tumors was greater than 3, LR was also performed. LT was performed in patients with diffuse diseases but without imaging evidence of extrahepatic metastasis, regardless of liver function.

Surgical treatment for patients was contraindicated in the presence of diffuse diseases, extrahepatic metastasis, and severe liver functional impairment. Among these patients, ST was recommended for patients with Child–Pugh A/B liver function, while patients with severe liver function impairment were included in the surveillance group since they were unable to tolerate anti-tumor drugs. However, some patients with Child–Pugh A/B grade of liver function were reluctant to receive any treatment, and they were included in the surveillance group.

Groups were compared for sociodemographic characteristics, clinicopathologic characteristics, treatment strategies, and overall survival (OS). Survival was assessed from the time of initial treatment to the date of death. For patients without any further treatment, the date of pathological diagnosis was taken as the starting time, and the pathological diagnosis was based on fine needle aspiration (FNA) and small biopsy followed by IHC staining of the collected sample. The lung metastasis was detected by CT and confirmed by FNA. Date of death was obtained from medical records or telephone interview. The last follow-up date was 31 July 2022.

Uninodular disease was defined as having only one lesion, multinodular disease was defined as having two or three nodules, and if the number of nodules was greater than or equal to 4, the patients were classified as having diffuse disease. Peripheral or subcapsular growth was defined as at least one lesion in contact with the hepatic surface. Hepatic capsular retraction was defined as at least one lesion with capsular retraction. Macrovascular invasion (MaVI) was defined as the presence of at least one of the following tumor thrombus: portal vein (PV) tumor thrombus (PVTT), hepatic vein (HV) tumor thrombus (HVTT), or retro hepatic inferior vena cava (RHIVC) tumor thrombus (IVCTT).

### Statistical analysis

The cutoff value of age group for the survival analysis and multivariate analysis with Cox regression model was 60 years old. For the maximum diameter of the largest tumor (MDLT), we selected less than or equal to 3 cm, greater than 3 cm but less than or equal to 5 cm, greater than 5 cm but less than or equal to 10 cm, and greater than 10 cm as the cutoff value.

We entered and verified the data using commercially available statistical software SPSS (version 26.0; SPSS Inc, Chicago, Illinois, USA). We performed log-rank (Cox–Mantel) survival analyses using Kaplan–Meier methods to test differences in survival (in months) between patients in different groups. Univariate Cox regression analyses and multivariate proportional hazards regression model were carried out to identify independent prognostic factors. A *p* value of <0.05 was considered statistically significant for all analyses.

## Results

### Demographic characteristics and clinical presentation

**Figure 1:**
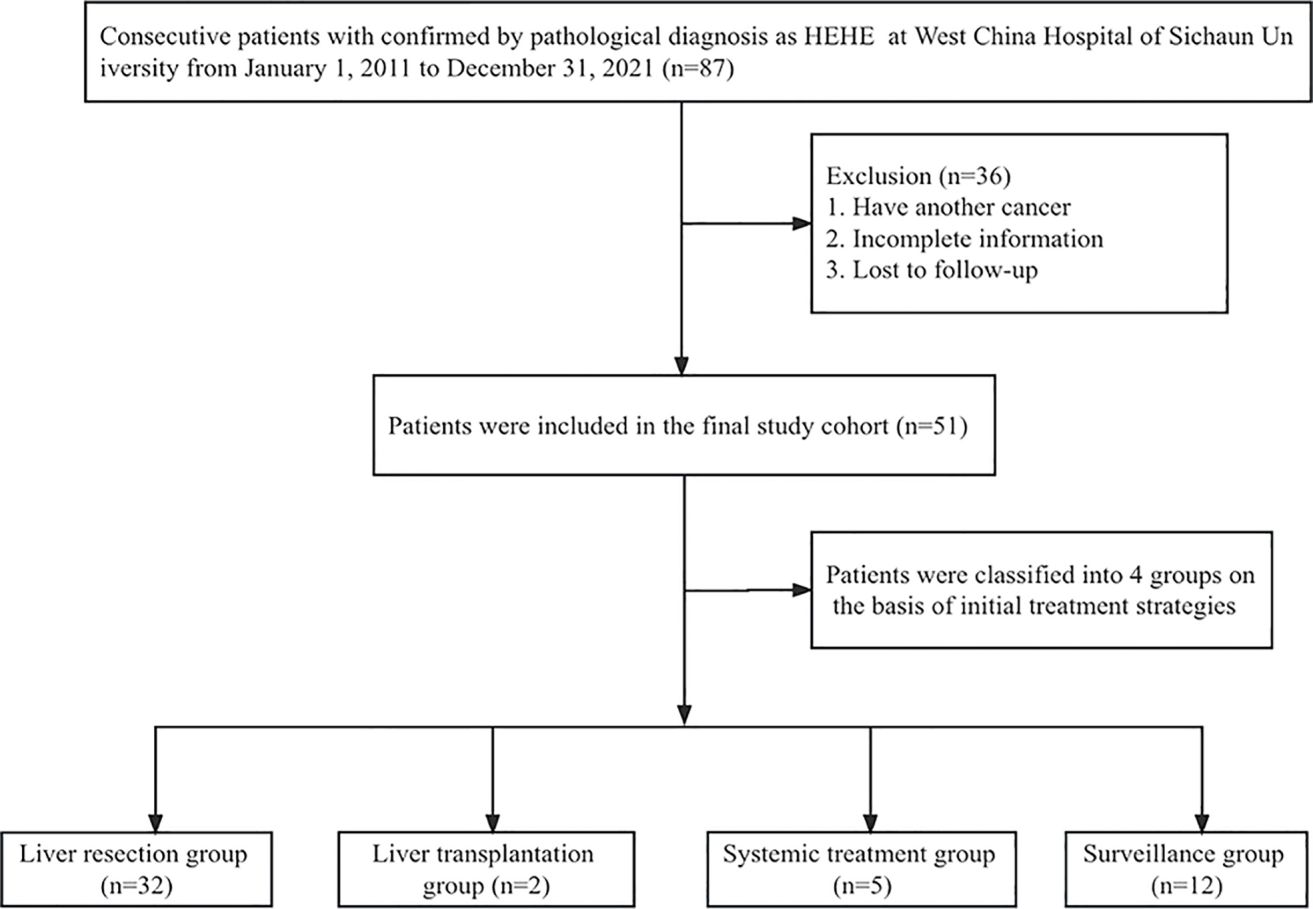
Flowchart of the study.

A total of 87 patients had a pathologic diagnosis of HEHE and thus were identified in the patient reports of West China Hospital of Sichuan University, but only 51 of them had complete available information (demographic, clinical data, and follow-up) and were included in the analysis (flowchart of the study, **Figure 1**). **Table 1** describes the baseline characteristics of this cohort. The majority of patients were women (*n* = 30), and the median age at diagnosis was 42.4 ± 11.4 years (range, 23 years to 69 years). The diagnosis of HEHE was incidental in 12 cases. The most frequent symptom in symptomatic patients was right upper quadrant and/or epigastrium pain (*n* = 24). Other symptoms include weight loss, nausea, anorexia, weakness, ascites, fever of unknown origin, and jaundice.

**Table 1:** Baseline characteristics of the patients with HEHE.

Variables	No. of patients
Age (years)	42.4 ± 11.4
Male/Female	21/30
Symptoms at diagnosis, yes/no	39/12
Type of symptoms^*^	
Abdominal pain in the right upper quadrant	24
Weight loss	5
Nausea	6
Anorexia	5
Weakness	2
Ascites	11
Fever of unknown origin	2
Jaundice	2
Anamnesis	
Oral contraceptive use	0
Trauma	2
Exposure to vinyl chloride	0
Exposure to polyurethane	0
Exposure to asbestos	0
Exposure to or silica	0
Primary biliary cirrhosis	0
CHB	2
Alcohol use	5

HEHE, hepatic epithelioid hemangioendothelioma; No., number; CHB, chronic viral hepatitis B.

All patients denied any history of exposure to vinyl chloride, polyurethane, asbestos, or silica. None of them had used oral contraceptives. Two patients had a history of upper abdominal trauma with laceration of liver, and chronic viral hepatitis B (CHB) was detected in two patients. In addition, five patients have a history of alcohol use (**Table 1**).

### Imaging findings

Generally, HEHE manifested as uninodular, multinodular, or diffuse diseases with ill-defined margins. Five patients presented extrahepatic disease at diagnosis, all with lung metastasis. MaVI was found in 21 patients while lymph node involvements were not observed. Nodules were peripheral or subcapsular growth in most cases (*n* = 49). The target sign, lollipop sign, tumor vessel sign, and calcifications were found in 9 patients, 9 patients, 1 patient, and 13 patients, respectively. Only in eight patients was hepatic capsule retraction reported (**Table 2**). The most frequent imaging presentation was multinodular disease in 25 patients, followed by uninodular disease in 14 and diffuse disease in 12 patients (**Table 3**).

**Table 2:** Characteristics of the HEHE tumors according to different ancillary examination.

Variables	No. of patients, *n* (%)					
	CUS, *n* = 19	CEUS, *n* = 10	Contrast-enhanced CT, *n* = 37	Contrast-enhanced MRI, *n* = 19	Gd-EOB-DTPA-enhanced MRI, *n* = 5	Total, *n* = 51
Peripheral or subcapsular growth	17 (89.5%)	9 (90.0%)	36 (92.3%)	17 (89.5%)	4 (80.0%)	49 (96.1%)
Extrahepatic diseases	0	0	5 (13.5%) ^a^	0	0	5 (9.8%)
Target sign	0	0	6 (15.4%)	4 (21.1%)	1 (20.0%)	9 (17.6%)
Lollipop sign	0	0	6 (15.4%)	5 (26.3%)	1 (20.0%)	9 (17.6%)
Calcifications	4 (21.1%)	2 (20.0%)	13 (33.3%)	0	0	13 (25.5%)
Tumor vessel sign	0	0	0	0	1 (20.0%)	1 (2.0%)
Hepatic capsular retraction	0	0	7 (17.9%)	3 (15.8%)	0	8 (15.7%)
MaVI	0	0	17 (43.6%)	7 (36.8%)	3 (60.0%)	21 (41.2%)

HEHE, hepatic epithelioid hemangioendothelioma; No., number; CUS, conventional ultrasonography; CEUS, contrast-enhanced ultrasonography; CT, computed tomography; MRI, magnetic resonance imaging; Gd-EOB-DTPA-enhanced MRI, gadolinium ethoxybenzyl diethylenetriamine pentaacetic acid-enhanced MRI; MaVI, macrovascular invasion; CTPV, cavernous transformation of portal vein; PVT, portal vein thrombosis.

^a^ all extrahepatic diseases occurred in the lung and were detected by CT examination and confirmed by FNA.

**Table 3:** Baseline characteristics of the HEHE tumors according to the first treatment received.

HEHE characteristics	LR, *n* = 32	LT, *n* = 2	ST, *n* = 5	Surveillance, *n* = 12	All, *n* = 51
Uninodular disease, *n* (%)	12 (37.5%)	0	1 (20.0%)	1 (8.3%)	14 (27.5%)
Multinodular disease, *n* (%)	20 (62.5%)	0	1 (20.0%)	4 (33.3%)	25 (49.0%)
Diffuse disease, *n* (%)	0	2 (100.0%)	3 (60.0%)	7 (58.3%)	12 (23.5%)
Lymph node involvement, *n* (%)	0	0	0	0	0
MaVI, *n* (%)	9 (28.1%)	1 (50.0%)	1 (20.0%)	10 (83.3%)	21 (41.2%)
Lung metastasis *n* (%)	0	0	1 (20.0%)	4 (33.3%)	5 (9.8%)
Child–Pugh A/B	32 (100%)	1 (50.0%)	5 (100.0%)	10 (75.0%)	44 (86.3%)
Child–Pugh C	0	1 (50.0%)	0	2 (25.0%)	4 (7.8%)

HEHE, hepatic epithelioid hemangioendothelioma; LR, liver resection; LT, liver transplantation; ST, system treatment; MaVI, macrovascular invasion.

#### CUS and CEUS

Images of CUS and CEUS were retrospectively evaluated by two independent abdominal ultrasonography physicians in consensus. HEHE criteria evaluated included number of lesions, maximum diameter, echogenicity (hyperechoic, hypoechoic, or isoechoic), homogeneous or heterogeneous, shape (regular or lobulated), margin (ill- or well-defined appearance), and color Doppler imaging features.

**Figure 2:**
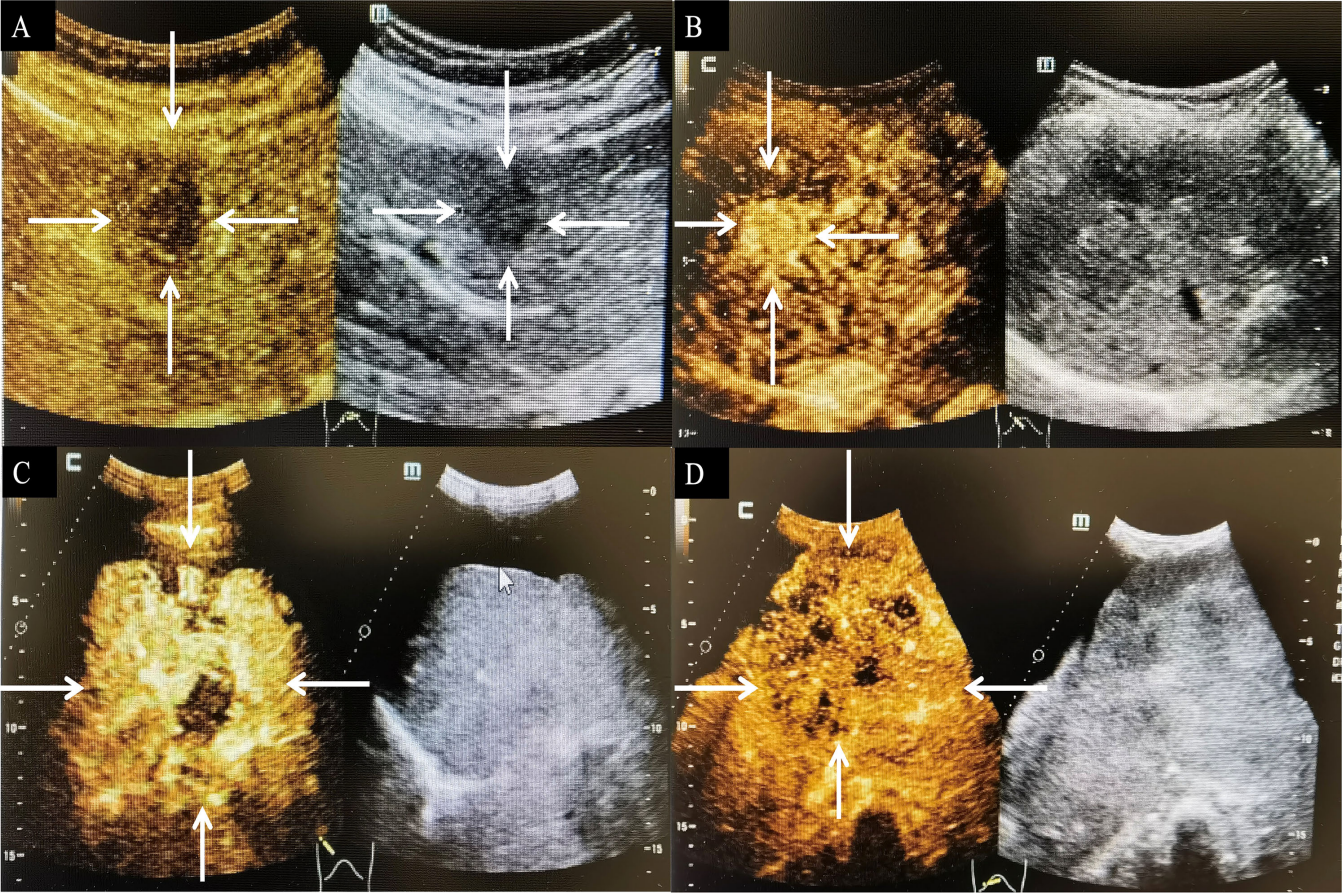
CEUS feature of HEHE. A 53-year-old female patient with hypoechoic nodule in the right lobe of the liver (arrows) **(A)** and a wholly uniformly enhanced nodule in the left lateral lobe (arrows) **(B)**. A 33-year-old male patient with heterogeneous hyperenhancement at the arterial phase (arrows) **(C)** and hypoenhancement of the central part at portal and late phases (arrows) **(D)**. CEUS, contrast-enhanced ultrasonography; HEHE, hepatic epithelioid hemangioendothelioma.

Nineteen patients had been evaluated by CUS. In CUS, either discrete nodules or diffusely echotexture regions may be seen. Most of the lesions were located in the peripheral area of the liver (*n* = 17). The lesions were mainly hypoechoic (*n* = 17) to adjacent liver parenchyma (**Figure 2A**), and heterogeneous echogenicity with hyperechoic focal liver lesion was also observed (*n* = 1). Hepatic capsular retraction was not obvious on CUS or CEUS, while calcifications were found in four patients.

Ten patients received CEUS. In CEUS, most of the lesions were located in the peripheral area of the liver (*n* = 9). All patients showed contrast agent dilution and presented hypoenhancement in the portal and late phase. HEHE presented peripheral nodular enhancement (*n* = 5) or heterogeneous hyperenhancement (*n* = 5) at the arterial phase (**Figure 2C**) and hypoenhancement (*n* = 10) at the portal and late phases (**Figure 2D**). The presence of central irregular nonenhancement zones was observed in the lesions of seven patients at all phases. A total of 33 lesions were evaluated by CEUS, most of which were peripheral or subcapsular growth (*n* = 29). Lesions ≤ 2 cm (*n* = 10) were mostly (*n* = 8) uniformly enhanced as a whole (**Figure 2B**), while lesions > 2 cm (*n* = 23) were mostly (*n* = 21) peripheral enhancement with no or low enhancement of the central part (**Figure 2C**). Calcifications within the nodules was recognizable in two patients.

#### Contrast-enhanced CT, contrast-enhanced MRI, Gd-EOB-DTPA-enhanced MRI, and ^18^F−FDG PET/CT

Images of contrast-enhanced CT, contrast-enhanced MRI, Gd-EOB-DTPA-enhanced MRI, and ^18^F−FDG PET/CT were retrospectively evaluated by two independent abdominal radiologists in consensus. The number, size, and location of the lesion were reported. For multifocal tumors, the location and largest axial dimension of the dominant lesion were recorded. The presence of suggestive features, such as coalescence of multifocal tumors, lollipop sign, target sign, and hepatic capsular retraction, was noted.

**Figure 3:**
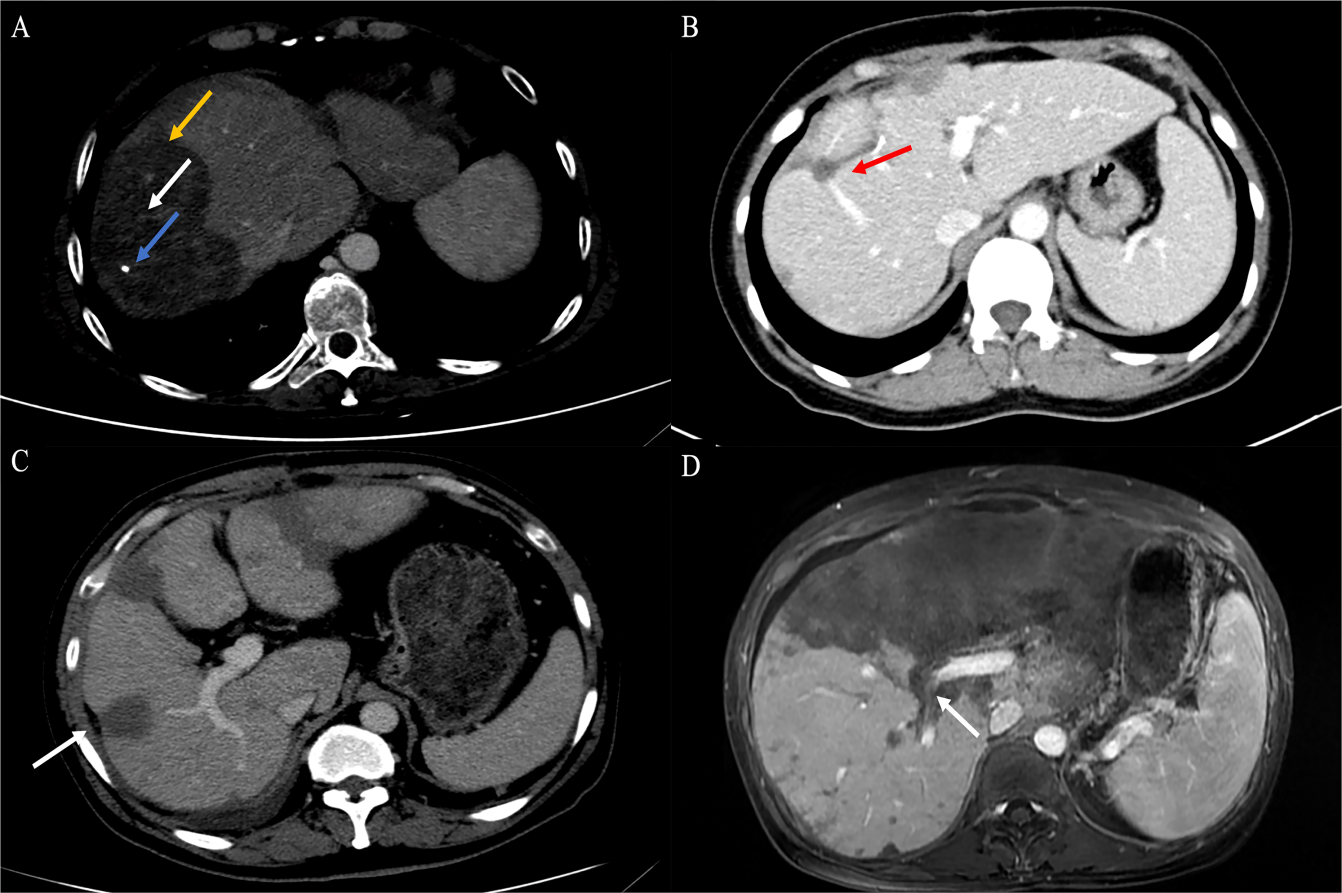
Findings of HEHE in contrast-enhanced CT. **(A)** Portal phase image contrast-enhanced CT demonstrated a large peripherally located lesion with calcification (blue arrow). Although the lesion was predominantly hypodense, there was a subtle non-enhancing central core (white arrow), surrounded by minimally enhancing peripheral layer (orange arrow). **(B)** The peripheral lesion demonstrated abrupt “cutoff” of a tributary of the RHV at the edge of a well-defined hypodense area; an example of the lollipop sign (red arrow). **(C)** Image of contrast-enhanced CT showed numerous variable-size hypodense nodules with hepatic capsular retraction (white arrow). **(D)** Contrast-enhanced CT showed diffuse coalescing hypoattenuating lesions; RPV and right posterior PV have been seen passing through the middle of the lesions (white arrow). CT, computed tomography; RHV, right hepatic vein; RPV, right branch portal vein; PV, portal vein.

**Figure 4:**
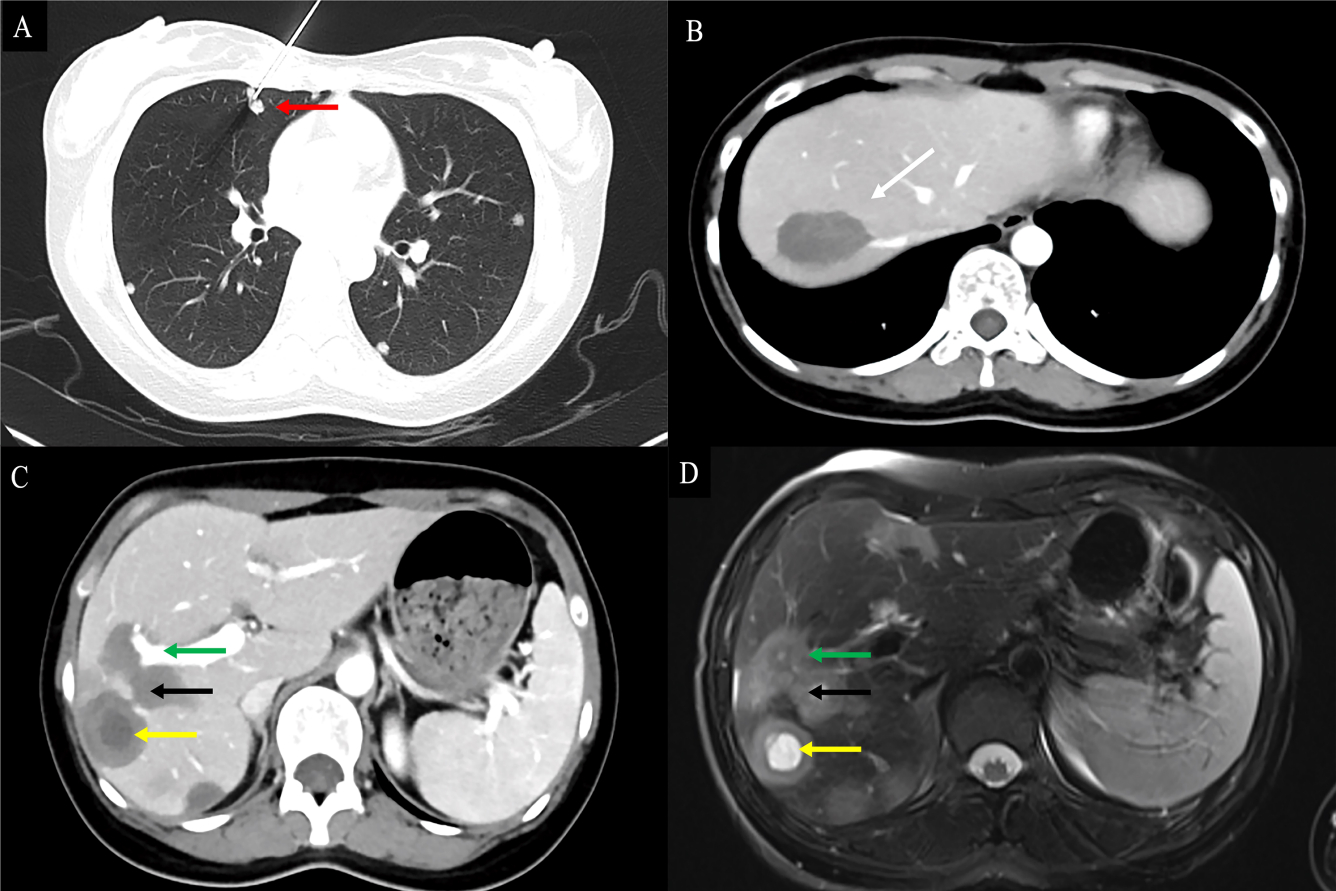
Findings of HEHE in contrast-enhanced CT and contrast-enhanced MRI. A 26-year-old female patient with HEHE was undergoing CT-guided FNA **(A)**. The image of abdominal contrast-enhanced CT demonstrated a peripherally located characteristic “lollipop sign” (white arrow) **(B)** and a “target sign” (yellow arrow) associated with invasion of the RPV (green arrow) and coalescent lesions (black arrow) **(C)**. The corresponding T2WI demonstrated the characteristic “target sign” (yellow arrow) with coalescent lesions (black arrow) **(D)**. HEHE, hepatic epithelioid hemangioendothelioma; CT, computed tomography; MRI, magnetic resonance imaging; FNA, fine needle aspiration; RPV, right branch portal vein; T2WI, T2-weighted image.

Contrast-enhanced CT was performed in 39 patients. Tumors were predominantly located in the peripheral, subcapsular regions of the liver (*n* = 36). The appearance of calcification (**Figure 3A**), lollipop sign (**Figures 3B**, **4B**), hepatic capsular retraction (**Figure 3C**), and target sign (**Figure 4C**) was seen in 13 patients, 6 patients, 7 patients, and 6 patients, respectively. MaVI was present in 17 patients (**Figure 3D**). Tumors were coalescent in 24 cases (**Figures 3C**, **4C**). Assessment of the dynamic CT revealed peripheral ring enhancement on arterial phase imaging in 12 patients. Low-density pattern was the most common abnormal feature and was found in 37 patients. High-density and heterogeneous mixed-density lesions were found in one patient for each patten. The majority (*n* = 32) of patients had enhancement, whereas no enhancement (*n* = 5) and variable, irregular enhancement (*n* = 2) were also reported. Lung metastasis was detected in five patients and was confirmed by FNA (**Figure 4A**). It was noteworthy that accurate diagnoses were reported only in 3 patients, 19 patients were misdiagnosed as MCA, 9 patients were misdiagnosed as HAS, 4 patients were misdiagnosed as HHA, 3 patients were misdiagnosed as ICC, and 1 patient was misdiagnosed as liver fibrosis with regenerative nodules caused by the abnormal development of hepatic vascular tissue.

**Figure 5:**
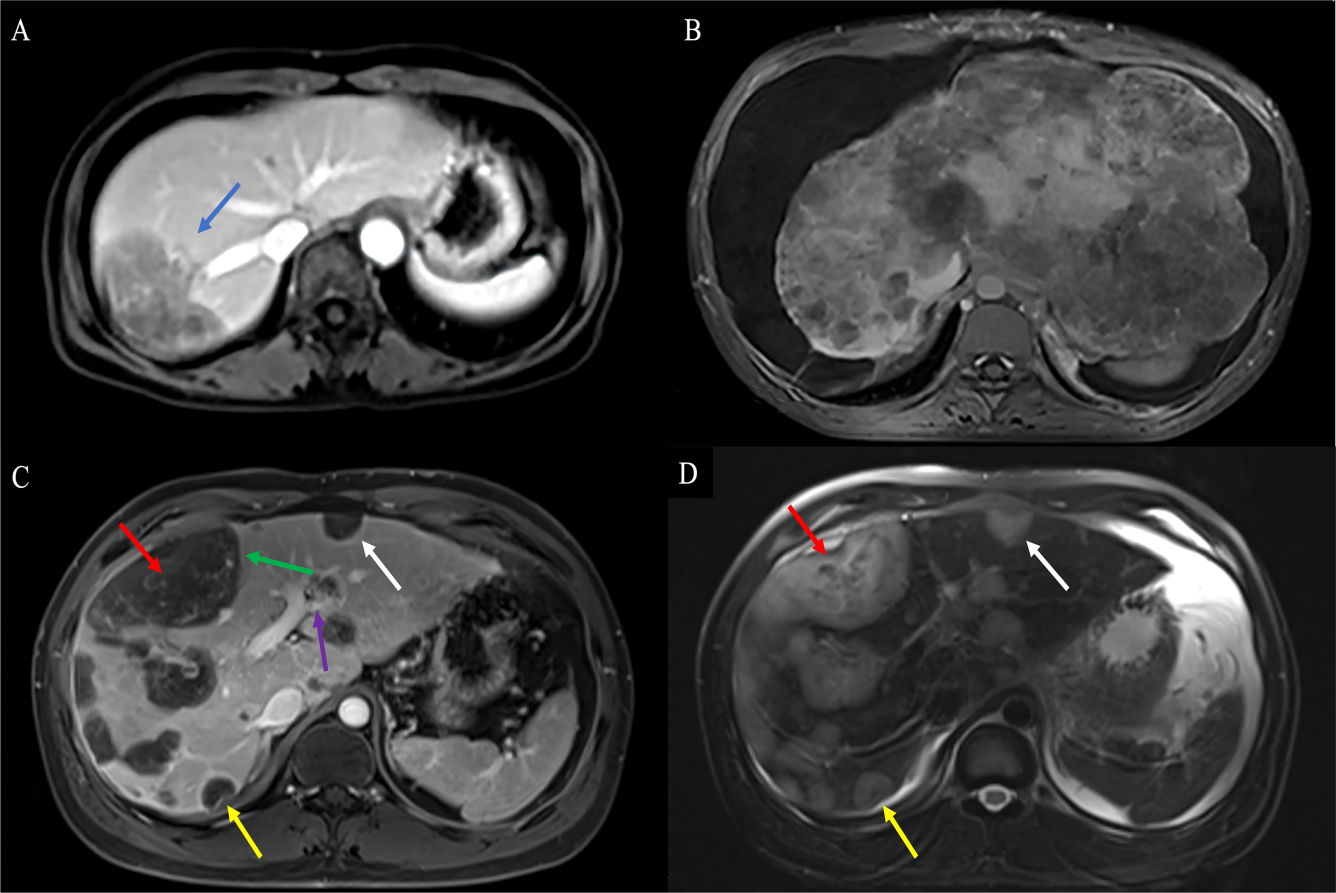
Findings of HEHE in contrast-enhanced MRI. **(A)** T1WI of contrast-enhanced MRI demonstrated a uninodular tumor with the characteristic “lollipop sign” (blue arrow). **(B)** T1WI of contrast-enhanced MRI demonstrated extremely a heterogeneous enhancement diffuse hypo-signal of varying sizes with hepatomegaly, RPV, and RHIVC compression. **(C)** Axial T1WI demonstrated predominantly peripherally located central hypointensity (red arrow) with peripheral high-signal rim (green arrow) and characteristic hepatic capsular retraction (yellow arrow). The LPV was infiltrated (purple arrow). Smaller lesions demonstrated uniform hypointensity with clear margins (white arrow), while larger lesions showed heterogeneous enhancement hypointensity (red arrow) **(D)**. Axial fat-suppressed T2WI demonstrated hyperintensity in tumor. Smaller lesions showed uniform hyperintensity (white arrow), while larger lesions showed heterogeneous enhancement hyperintensity (red arrow). HEHE, hepatic epithelioid hemangioendothelioma; MRI, magnetic resonance imaging; T1WI, T1-weighted image; RPV, right branch portal vein; RHIVC, retro hepatic inferior vena cava; T2WI, T2-weighted image.

Contrast-enhanced MRI studies of the abdomen were available for 19 patients. HEHE manifested as a uninodular (**Figure 5A**), multinodular, or diffuse disease with most of the lesions located in the peripheral area of the liver (**Figure 5**). MaVI and hepatic capsular retraction were present in seven patients and three patients (**Figures 5C, D**), respectively. The lollipop sign (**Figure 5A**) and target sign (**Figure 4D**) were found in five patients and four patients, respectively. The tumor vessel sign was present in one patient. Tumors were coalescent in 11 cases (**Figures 5C, D**).

On T1-weighted images (T1WI), low signal intensity, low signal intensity with a peripheral dark rim, and iso-signal intensity with a peripheral dark rim were reported (**Figure 5C**). On T2-weighted images (T2WI), high signal intensity was the most frequent signal feature, followed by mixed signal intensity with a peripheral dark rim, high signal intensity with a peripheral dark rim, and central low signal intensity with a peripheral high-signal rim (**Figure 5D**).

Accurate diagnoses were reported only in two patients, eight patients were misdiagnosed as MCA, two patients were misdiagnosed as HAS, two patients were misdiagnosed as HHA, four patients were misdiagnosed as ICC, and one patient was misdiagnosed as fibrolamellar or sclerosing form of hepatocellular carcinoma (sHCC).

**Figure 6:**
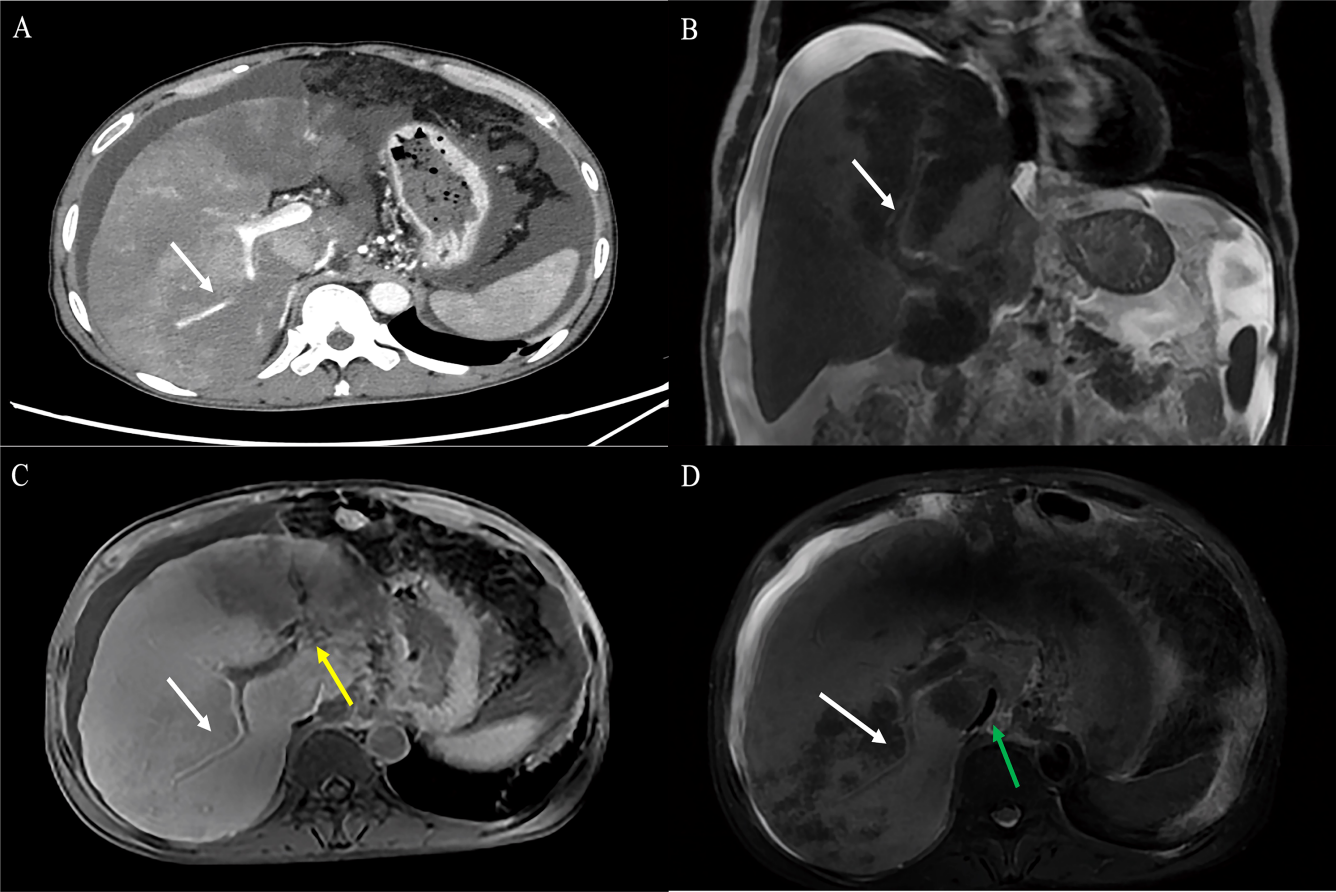
A 55-year-old male HEHE patient with the characteristic “tumor vessel sign”. **(A)** Axial contrast-enhanced CT demonstrated stenosis of the LPV and the right anterior branch of PV, with the right posterior portal vein passing through the tumor (white arrow). **(B)** The corresponding coronal Gd-EOB-DTPA-enhanced MRI demonstrated the right anterior branch of portal vein passing through the tumor with extensive tumor thrombus (white arrow). **(C)** The Gd-EOB-DTPA-enhanced MRI showed that the vessel was soft and naturally tapered, similar to normal vessels (white arrow) combined with cavernous transformation of portal vein (yellow arrow) and **(D)** RHIVC compression (green arrow). HEHE, hepatic epithelioid hemangioendothelioma; CT, computed tomography; LPV, left branch of portal vein; Gd-EOB-DTPA, Gadolinium ethoxybenzyl diethylenetriamine pentaacetic acid; RHIVC, retro hepatic inferior vena cava.

**Figure 7:**
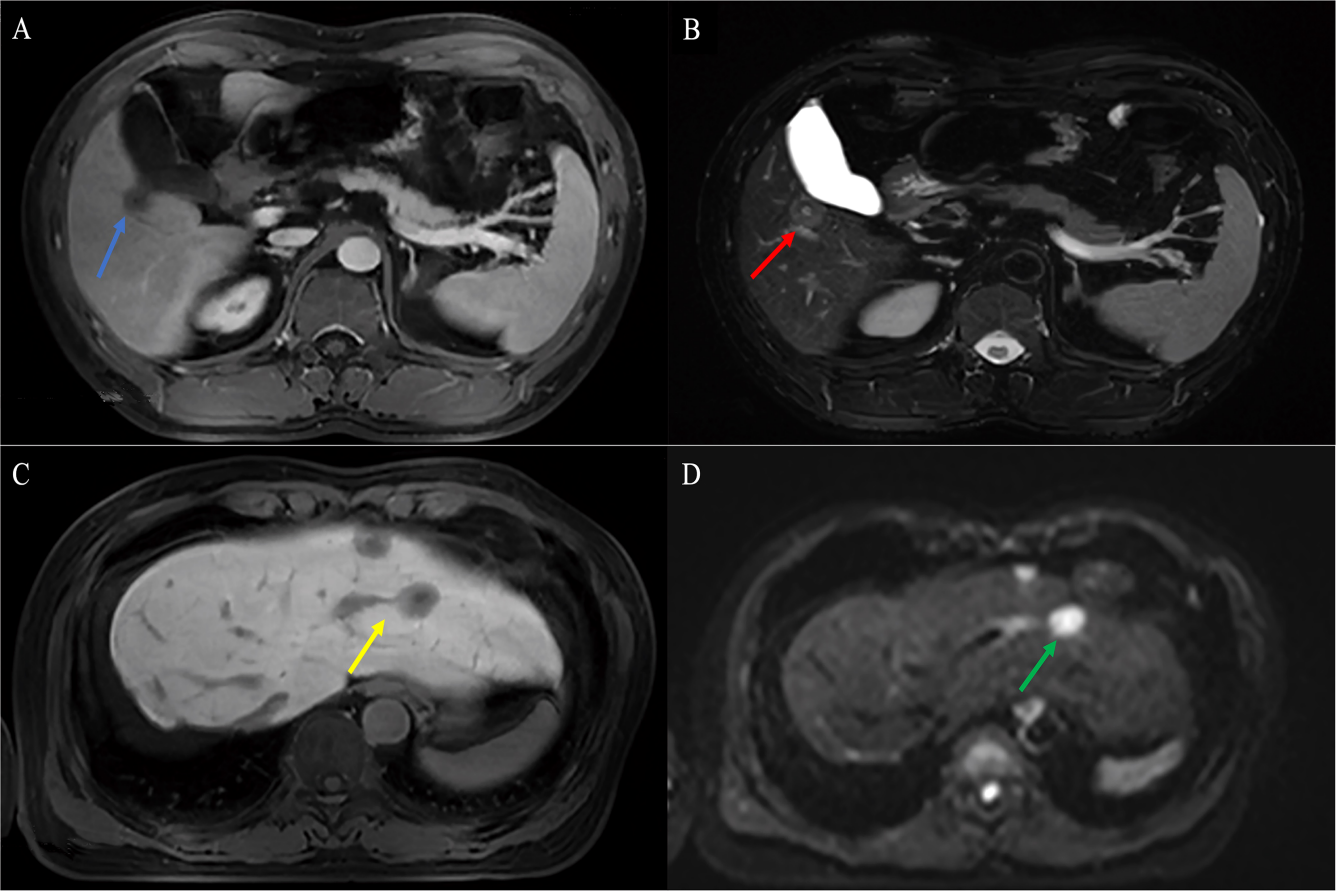
Findings of HEHE in Gd-EOB-DTPA-enhanced MRI. A 38-year-old male patient with a uninodular tumor about 1.8 cm in diameter with slight hypointensity on T1WI (blue arrow) **(A)** and hyperintensity in the center and outer layer, and hypointensity in the middle layer on T2WI (target sign, yellow arrow) **(B)**. A 50-year-old female patient with well-defined nodules in segment II and IV with hypointense and clear margins on T1WI (yellow arrow) **(C)**. Axial MRI demonstrated limited diffusion hyperintensity on diffusion-weighted imaging (DWI) (green arrow) **(D)**. HEHE, hepatic epithelioid hemangioendothelioma; Gd-EOB-DTPA, Gadolinium ethoxybenzyl diethylenetriamine pentaacetic acid; T1WI, T1-weighted image; T2WI, T2-weighted image; DWI, diffusion-weighted imaging.

The Gd-EOB-DTPA-enhanced MRI was obtained in five patients and resulted in high enhancement (*n* = 3) and peripheral and delayed central enhancement (*n* = 2). Gd-EOB-DTPA-enhanced MRI demonstrated lesions with decreases in Gd-EOB-DTPA uptake. The tumor vessel sign (**Figure 6**), target sign (**Figures 7A, B**), and lollipop sign (**Figures 7C, D**) were present in one patient each. Hepatic capsular retraction and tumor coalescence were not detected while MaVI was present in three patients (**Figure 6D**). The Gd-EOB-DTPA-enhanced MRI failed to give an accurate diagnosis of HEHE, while three patients were misdiagnosed as MCA, one patient was misdiagnosed as HAS, and one patient was misdiagnosed as sHCC.

**Figure 8:**
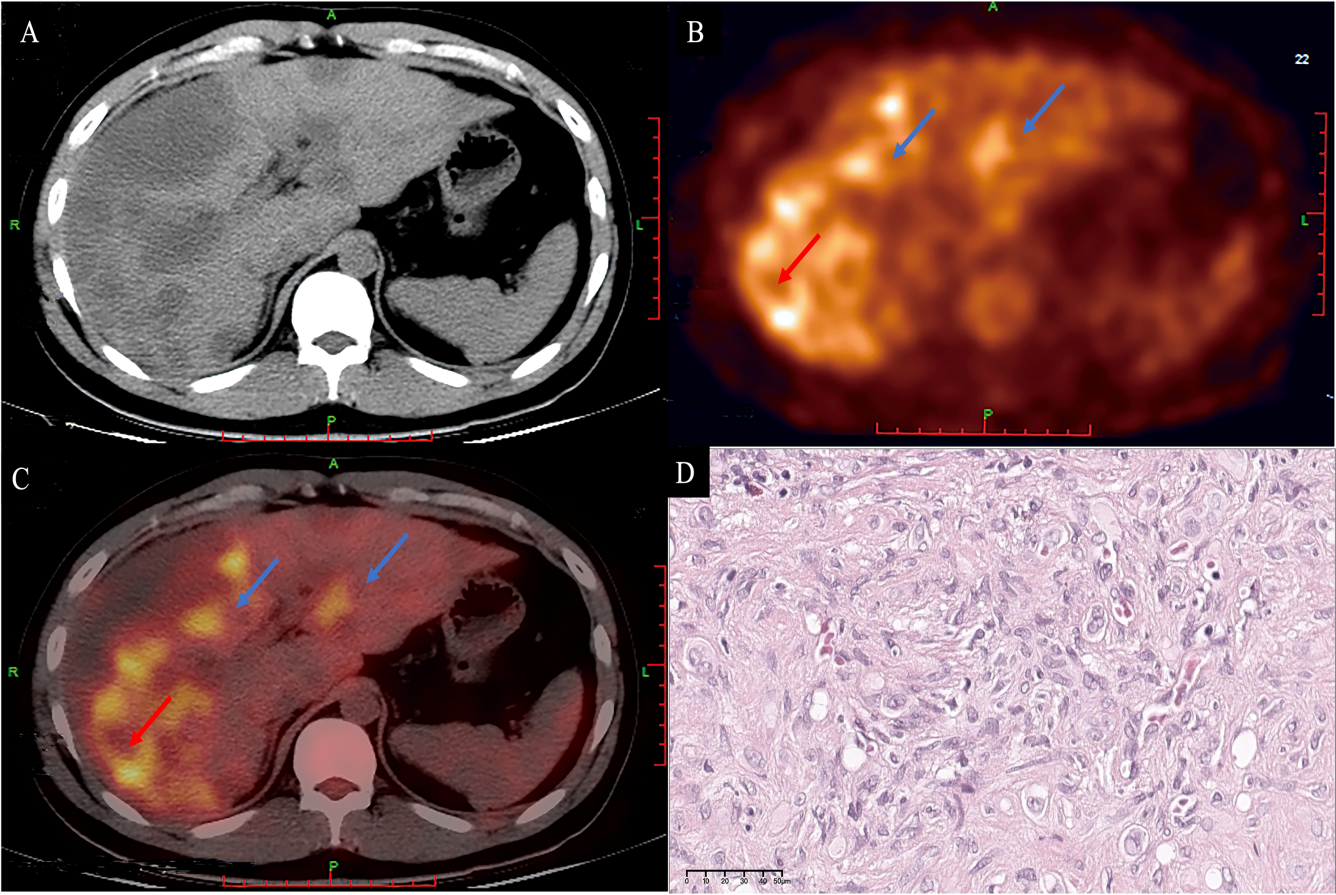
A 33-year-old male patient with diffuse HEHE. Axial contrast-enhanced CT demonstrated predominantly peripherally located hypointensity **(A)**. Corresponding ^18^F−FDG PET/CT **(B)** and fusion images **(C)** show that both the large and small lesions were hypermetabolic (blue arrows). The large lesion (red arrow) demonstrated a hypermetabolic peripheral rim with an SUV max of 6.59, and a relatively hypometabolic central area. Microscopically, the periphery of the tumor has high cellularity **(D)** (H&E, original magnification ×400). HEHE, hepatic epithelioid hemangioendothelioma; CT, computed tomography; ^18^F−FDG PET/CT, 2-[^18^F] fluoro-2-deoxy-D-glucose positron emission tomography/computed tomography.

The technique of ^18^F−FDG PET/CT has also been used in one patient with diffuse disease, and increased fluoro-deoxy-glucose (FDG) uptake above the background liver parenchyma was seen (**Figure 8**).

### Pathological features

**Figure 9:**
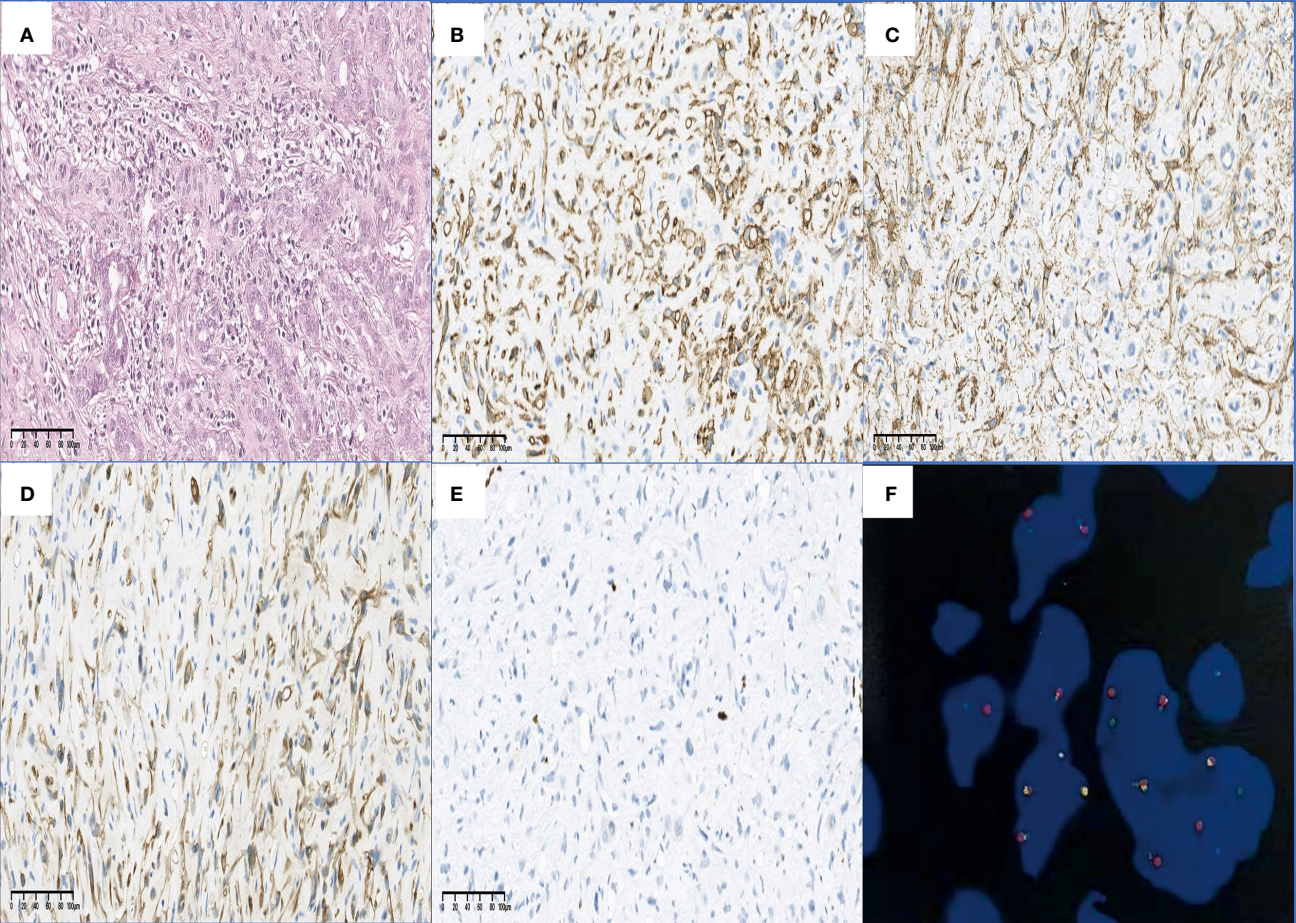
Findings of H&E, IHC staining, and FISH. HEHE cells tend to grow along vascular structures and infiltrate hepatic sinuses, causing hepatocyte atrophy and replacement **(A)** (H&E staining, original magnification ×200). The positive staining of CD34 **(B)** and SMA **(C)** in the HEHE cytomembrane demonstrated the formation of vascular structures and cytoskeleton structures in the primitive stage. In some cases, cytoplasmic positive CK7 **(D)** and nuclear positive Ki-67 **(E)** have also been seen (all IHC staining, original magnification ×200). A characteristic WWTR1-CAMTA1 fusion was identified by FISH **(F)**. H&E, hematoxylin–eosin; IHC, immunohistochemical; FISH, fluorescence *in situ* hybridization; CD, cluster of differentiation; SMA, smooth muscle actin; HEHE, hepatic epithelioid hemangioendothelioma; CK, cytokeratin; WWTR1-CAMTA1, WW domain-containing transcription regulator 1 with calmodulin-binding transcription activator 1.

In hematoxylin–eosin (H&E) staining, HEHE cells tend to grow along vascular structures and infiltrate hepatic sinuses, causing hepatocyte atrophy and replacement (**Figure 9A**). A large portion of tumor cells display an invasive growth pattern consisting of dendritic, epithelioid, and intermediate cells interspersed in a dense mucopolysaccharide extracellular matrix rich in hyaluronic acid. Despite the destruction of the liver plate, the portal vein and terminal hepatic venules remain intact. Some cells have an appearance like a signet ring with intracytoplasmic vacuoles or lumina.

In IHC staining, the vascular endothelial cell marker CD34 was positive in all the 51 patients (51/51) (**Figure 9B**), as well as CD31 in 49 cases (49/49), ERG in 44 patients (44/44), followed by FVIII in 26 of 29 cases (26/29) and Fli-1 in 12 of 14 cases (12/14). D2-40 was positive in 2 out of 7 cases (2/7). Epithelial membrane antigen (EMA) was found positive in 3 out of 36 patients (3/36), smooth muscle actin (SMA) was found positive in 2 out of 5 patients (2/5) (**Figure 9C**). Nuclear calmodulin-binding transcription activator 1 (CAMTA1) expression was observed in 88.5% of cases (23/26), while nuclear TFE3 expression was observed in 10.7% of cases (3/28). Twenty-one out of 43 cases (21/43) were positive for cytokeratin (CK), and CK18 was found positive in 2 out of 3 patients (2/3), followed by CK7 (9/17) (**Figure 9D**), CK8 (4/11), and CK19 (1/10). Tumor proliferation was graded according to Ki-67 positivity as low index [>0 and <10 positive nuclei/10 high power field (HPF)] in 23 of 27 cases (23/27) and as high index (≥10 positive nuclei/10 HPF) in 4 cases (4/27) (**Figure 9E**).

Fluorescence *in situ* hybridization (FISH) analysis was undertaken in six patients to explore the presence of rearrangements in the previously documented 1p36 and 3q25 chromosomal regions. Further molecular characterization has identified a gene fusion of WWTR1 (WW domain-containing transcription regulator 1) with CAMTA1 (calmodulin-binding transcription activator 1) (WWTR1-CAMTA1) in all of them (**Figure 9F**).

A summary of IHC profiles is shown in **Figure 9** and **Table 4**.

**Table 4:** Immunohistochemical analyses.

Case	CAMTA1	WWTR-CAMTA1	TFE3	FVIII	CD31	CD34	ERG	FLI-1	EMA	CK8	CK18	CK7	CK19	PCK	D2-40	SMA
1	–	N/A	–	N/A	+	+	+	N/A	N/A	N/A	N/A	+	–	–	N/A	N/A
2	N/A	N/A	N/A	N/A	+	+	+	N/A	N/A	–	N/A	–	–		N/A	N/A
3	N/A	N/A	N/A	+	+	+	N/A	+	N/A	N/A	N/A	–	–	–	N/A	N/A
4	+	N/A	–	N/A	+	+	+	N/A	N/A	N/A	N/A	–	–	–	N/A	N/A
5	N/A	N/A	N/A	N/A	+	+	+	N/A	N/A	–	N/A	N/A	–	N/A	N/A	N/A
6	N/A	N/A	N/A	N/A	+	+	+	–	–	N/A	N/A	N/A	–	–	N/A	N/A
7	N/A	N/A	N/A	N/A	+	+	+	N/A	–	N/A	N/A	N/A	–	–	N/A	N/A
8	+	N/A	N/A	+	+	+	+	N/A	–	N/A	N/A	N/A	–	–	N/A	N/A
9	+	N/A	–	N/A	+	+	+	+	+	N/A	N/A	N/A	–	–	N/A	N/A
10	+	N/A	N/A	+	+	+	+	N/A	–	+	N/A	+	N/A	+	N/A	N/A
11	+	N/A	+	+	+	+	+	+	+	N/A	N/A	+	N/A	N/A	N/A	N/A
12	+	+	–	N/A	+	+	+	N/A	–	N/A	N/A	+	N/A	–	N/A	N/A
13	+	N/A	–	N/A	+	+	+	N/A	N/A	N/A	N/A	+	N/A	–	N/A	N/A
14	N/A	N/A	N/A	+	+	+	+	+	–	–	+	–	N/A		N/A	N/A
15	N/A	N/A	–	+	+	+	+	N/A	–	N/A	N/A	–	N/A	–	–	–
16	N/A	N/A	N/A	N/A	+	+	+	+	–	+	N/A	N/A	N/A	N/A	N/A	–
17	+	N/A	–	+	+	+	+	N/A	–	–	+	N/A	N/A	+	–	N/A
18	N/A	N/A	N/A	+	+	+	+	N/A	–	–	N/A	N/A	N/A	–	N/A	N/A
19	N/A	N/A	N/A	+	+	+	N/A	+	N/A	N/A	N/A	N/A	N/A	–	N/A	N/A
20	N/A	N/A	N/A	+	+	+		N/A	–	N/A	N/A	N/A	N/A	–	N/A	N/A
21	N/A	N/A	N/A	+	+	+	+	N/A	–	N/A	N/A	N/A	N/A	–	–	N/A
22	N/A	N/A	N/A	+	+	+	+	N/A	–	N/A	N/A	N/A	N/A	+	+	N/A
23	N/A	N/A	N/A	+	+	+	+	N/A	–	N/A	N/A	N/A	N/A	+	–	N/A
24	+	N/A	–	+	+	+	+	N/A	–	N/A	N/A	N/A	N/A	+	N/A	N/A
25	N/A	+	N/A	+	+	+	+	N/A	–	N/A	N/A	N/A	N/A	+	N/A	+
26	N/A	+	–	N/A	+	+	+	N/A	–	N/A	N/A	N/A	N/A	–	N/A	+
27	+	N/A	–	N/A	+	+	+	+	–	N/A	N/A	N/A	N/A	N/A	–	N/A
28	+	N/A	–	+	+	+	+	N/A	–	N/A	N/A	N/A	N/A	–	N/A	N/A
29	N/A	N/A	N/A	+	+	+	N/A	N/A	–	N/A	N/A	N/A	N/A	N/A	N/A	N/A
30	N/A	N/A	N/A	+	+	+	+	N/A	–	N/A	N/A	N/A	N/A	+	N/A	N/A
31	N/A	N/A	N/A	+	+	+	+	N/A	–	N/A	N/A	N/A	N/A	–	–	N/A
32	N/A	N/A	N/A	+	+	+	+	N/A	N/A	N/A	N/A	N/A	N/A	N/A	N/A	N/A
33	+	N/A	–	+	+	+	+	+	–	+	N/A	–	N/A	N/A	N/A	–
34	+	N/A	–	N/A	N/A	+	+	N/A	N/A	N/A	N/A	N/A	N/A	+	N/A	N/A
35	+	N/A	–	N/A	+	+	+	N/A	N/A	–	N/A	–	N/A	N/A	–	N/A
36	+	N/A	–	N/A	+	+	+	N/A	–	N/A	N/A	N/A	N/A	–	N/A	N/A
37	–	N/A	–	N/A	+	+	+	N/A	N/A	N/A	N/A	N/A	N/A	–	N/A	N/A
38	+	N/A	+	+	+	+	+	+	–	N/A	N/A	N/A	N/A	–	N/A	N/A
39	+	N/A	N/A	N/A	+	+	N/A	+	N/A	N/A	N/A	N/A	N/A	N/A	N/A	N/A
40	–	N/A	+	–	+	+	+	N/A	+	N/A	N/A	N/A	N/A	N/A	N/A	N/A
41	+	N/A	–	N/A	N/A	+	+	N/A	–	N/A	N/A	N/A	N/A	N/A	N/A	N/A
42	+	N/A	–	N/A	+	+	+	+	+	N/A	N/A	N/A	N/A	+	+	N/A
43	+	N/A	–	+	+	+	+	N/A	N/A	+	–	+	N/A	+	N/A	N/A
44	+	N/A	–	N/A	+	+	+	N/A	–	N/A	N/A	N/A	N/A	–	N/A	N/A
45	+	N/A	–	–	+	+	+	N/A	–	N/A	N/A	N/A	N/A	N/A	N/A	N/A
46	+	N/A	–	N/A	+	+	N/A	N/A	–	N/A	N/A	N/A	N/A	N/A	N/A	N/A
47	N/A	+	–	N/A	+	+	+	N/A	–	N/A	N/A	+	N/A	+	N/A	N/A
48	N/A	+	–	–	+	+	+	N/A	–	N/A	N/A	–	N/A	N/A	N/A	N/A
49	N/A	+	–	+	+	+	+	N/A	N/A	N/A	N/A	+	N/A	N/A	N/A	N/A
50	N/A	N/A	N/A	+	+	+	N/A	+	N/A	N/A	N/A	+	+	+	–	N/A
51	N/A	N/A	N/A	+	+	+	N/A	N/A	–	–	N/A	N/A	N/A	+	N/A	N/A

CAMTA1, calmodulin-binding transcription activator 1; WWTR-CAMTA1, WW domain-containing transcription regulator 1 with calmodulin-binding transcription activator 1; TFE3, transcription factor E3; FVIII, factor VIII-antigen; CD, cluster of differentiation; FLI-1, friend leukemia integration 1 transcription factor; EMA, epithelial membrane antigen; CK, cytokeratin; PCK, pan cytokeratin; SMA, smooth muscle actin; N/A, not done.

“+” means positive and “-” means negative.

### Treatment strategies and follow-up

Twelve patients did not receive any specific therapy and thus were included in the surveillance group. The other patients received at least one initial treatment (**Table 3**). After a median follow-up of 61.0 months, 22 patients developed at least one tumor progression or metastasized to other organs and 15 were died of disease (DOD).

Twelve of the 32 patients treated with LR had uninodular disease and 20 had multinodular disease. Nine patients had MaVI (**Table 3**). Six of the patients treated with LR developed tumor recurrence in the form of intrahepatic recurrence, while three patients developed lung metastasis after LR. At the last follow-up, 22 patients were still alive with no evidence of disease, 4 patients were alive with disease (AWD), and 6 were DOD. The survival time ranged from 3 to 116 months.

Two patients with diffuse disease underwent LT, one patient died of liver failure 1 month after LT, and another patient received a second LT 66 months later and was still alive 103 months after first LT.

Five patients received ST because of the presence of extrahepatic diseases and diffuse disease. The initial therapeutic agents were various, such as cis platinum combined with fluorouracil and calcium folinate (in two patients), Anlotinib (in two patients), and Bevacizumab combined with Sindilizumab and xeloda (in one patient). Two of them had more than one regimen during the course of therapy with secondary agents including itraconazole combined with Bevacizumab and xeloda (in one patient) and Anlotinib (in one patient). Transcatheter arterial chemoembolization (TACE) was not performed in this study. All patients treated with ST were monitored with CT and/or MRI every 3 to 6 months during treatment. One patient had lung metastasis 2 months after initial treatment, and another patient had lung metastasis and local progression 1 month after initial treatment. Three patients were AWD at the last follow-up and two were DOD.

Twelve patients received no treatment because of the presence of diffuse diseases, extrahepatic metastasis, and severe liver functional impairment, and some patients with Child–Pugh A/B grade of liver function were reluctant to receive treatment. At the last follow-up, six patients were AWD and six were DOD. Four out of these six patients were DOD within 3 months, one patient was DOD 8 months later, and the last one was DOD 84 months later after diagnosis. The survival time ranged from 1 to 107 months.

### Prognosis

After a median follow-up of 61.0 months, the 1-, 3-, and 5-year survival rates were 80.3%, 77.3%, and 67.1% in the whole study regardless of treatment. The 1-, 3-, and 5-year survival rates according to treatment groups were 92.2%, 90.1%, and 76.0% in LR patients; 50.0%, 50.0%, and 50% in LT patients; and 57.1%, 56.0%, and 42.3% in surveillance patients, respectively. The 1- and 3-year survival rates were 56.0% and 53.3% in those treated with ST, respectively. In addition, the 1-, 3-, and 5-year survival rates were 89.8%, 87.7%, and 74.7% in the surgical approach (LR+LT), and 57.9%, 56.5%, and 44.3% in the nonsurgical approach (ST+ surveillance), respectively.

The duration of follow-up ranged from 1 to 116 months. Thirty-six patients were alive at the last follow-up and 15 were DOD. Nineteen patients had survived more than 5 years and 17 of them were still alive at the last follow-up. Death was attributable to the neoplasm in all of the 15 patients and occurred between 1 and 84 months after diagnosis.

Because of the number of censored cases, median survival could not be calculated for the LR group, ST group, and surgical approach group. A significant difference was noted between the LR group and the surveillance group with respect to mean survival [95.98 (95% CI 81.76–110.21) vs. 56.00 (95% CI 28.10–83.91), months, *p* = 0.006], as was in the LR group and the ST group [95.98 (95% CI 81.76–110.21) vs. 27.00 (95% CI 10.43–43.57), months, *p* = 0.036]. The OS between the ST group and the surveillance group was not significantly different [27.00 (95% CI 10.43–43.57) vs. 56.00 (95% CI 28.10–83.91), months, *p* = 0.851]. A significant difference was noted between surgical approach (LR and LT) and nonsurgical approach (ST and surveillance) with respect to mean survival [93.95 (95% CI 79.55–108.36) vs. 56.06 (95% CI 32.07–80.05), months, *p* = 0.008].

**Figure 10:**
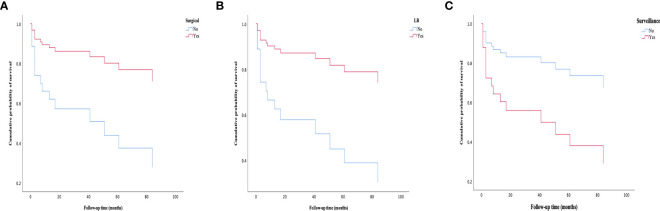
Survival curves of patients in different groups. **(A)** Patients treated with surgical approaches have a better survival rate than those treated with nonsurgical approaches. **(B)** Patients treated with LR have a better survival rate than those treated without LR. **(C)** Patients in the surveillance group have experienced a worse survival rate than those treated with active treatment (LR, LT or ST). LR, liver resection; LT, liver transplantation; ST, systemic treatment.

When we analyzed the prognostic factors of the tumors, we identified that LR [HR = 3.99 (95% CI 1.39–11.47), *p* = 0.010] and surgical approach [HR = 3.74 (95% CI 1.31–10.67), *p* = 0.014] as positive predictors of outcome, while MaVI [HR = 3.15 (95% CI 1.07–9.23), *p* = 0.037], lung metastasis [HR = 4.13 (95% CI 1.07–16.00), *p* = 0.040], and surveillance [HR = 3.15 (95% CI 1.10–9.00), *p* = 0.033] were identified as poor prognostic factors in univariate analysis. Multivariate analysis showed that LR [HR = 3.99 (95% CI 1.39–11.47), *p* = 0.010] and surgical approach [HR = 3.74 (95% CI 1.31–10.67), *p* = 0.014] were independently associated with good OS, while surveillance [HR = 3.15 (95% CI 1.10–9.00), *p* = 0.033] was independently associated with poor OS (**Figure 10**).

In addition, when adjusting for confounding factors, patients in the LR group have much better OS than those in the surveillance group [HR = 4.38 (95% CI 1.37–14.00), *p* = 0.013]. However, there was no significant difference in OS between the LR group and the ST group (*p* = 0.254), as was in the ST group and the surveillance group (*p* = 0.857) (**Table 5**).

**Table 5:** Univariate and multivariate analysis for overall survival.

	Univariate analysis		Multivariate analysis	
	HR (95% CI)	*p*-value	HR (95% CI)	*p*-value
Age group				
≥60	1.79 (0.23–13.73)	0.576	Not selected	Not selected
≥65	0.48 (0.06–3.77)	0.485	Not selected	Not selected
Gender	1.42 (0.51–3.91)	0.503	Not selected	Not selected
MDLT				
≤3 cm	2.40 (0.76–7.57)	0.135	Not selected	Not selected
>3 cm and ≤5 cm	0.51 (0.17–1.50)	0.219	Not selected	Not selected
>5 cm and ≤10 cm	1.74 (0.26–2.10)	0.574	Not selected	Not selected
>10 cm	0.26 (0.06–1.20)	0.085	Not selected	Not selected
The distribution pattern of tumors				
Uninodular disease	2.43 (0.54–10.82)	0.245	Not selected	Not selected
Multinodular disease	1.41 (0.41–3.16)	0.800	Not selected	Not selected
Diffuse disease	0.39 (0.13–1.14)	0.086	Not selected	Not selected
MaVI	3.15 (1.07–9.23)	0.037	–	–
Lung metastasis	4.13 (1.07–16.00)	0.040	–	–
Child–Pugh A/B grade of liver function	2.54 (0.32–20.27)	0.378	Not selected	Not selected
Treatment strategies				
LR	3.99 (1.39–11.47)	0.010	3.99 (1.39–11.47)	0.010
LT	0.54 (0.07–4.21)	0.558	Not selected	Not selected
ST	0.46 (0.10–2.12)	0.318	Not selected	Not selected
Surveillance	3.15 (1.10–9.00)	0.033	3.15 (1.10–9.00)	0.033
Surgical approach	3.74 (1.31–10.67)	0.014	3.74 (1.31–10.67)	0.014

HR, hazard ratio; CI, confidence interval; MDLT, maximum diameter of the largest tumor; cm, centimeter; MaVI, macrovascular invasion; LR, liver resection; LT, liver transplantation; ST, system treatment; MaVI, macrovascular invasion.

In summary, HEHE is a rare malignant tumor of vascular origin with unknown etiology and a variable natural course. Little is known about the pathophysiology, clinical course, and management of this disease because of its low incidence rate. The distribution pattern of HEHE can be characterized as uninodular, multinodular, or diffuse disease. It was not possible to make a specific diagnosis without biopsy in HEHE because the radiological findings were similar to those in some hepatic malignancies. There is no well-defined treatment strategy for HEHE. Compared with other malignant liver tumors, HEHE has a good prognosis.

## Discussion

Epithelioid hemangioendothelioma (EHE) is a rare low-grade malignant tumor of mesenchymal cell origin, characterized by the source of epithelioid cells and vascular endothelial tissue, with the characteristics of multifocal origin, that is, multiple organs occur simultaneously or successively, and it was difficult to distinguish between multiple primary lesions and MCA. Because of its rarity, HEHE has often been misdiagnosed as HHA, HAS, primary liver cancer, ICC, sHCC, or other diseases, and the preoperative diagnosis compliance rate was relatively low (6, 9).

The incidence and etiology of HEHE also remain unclear. Several possible pathogenic factors and risk factors have been identified such as exposure to vinyl chloride, polyurethane, asbestos, or silica; oral contraceptive use; primary biliary cirrhosis; viral hepatitis; and alcohol use (3).

HEHE most commonly occurs between the ages of 30 and 50 years, with a predominance in female patients based on a female-to-male incidence ratio of 1.5:1 (6). The clinical presentations were nonspecific, and 23.5% of the patients in our study were incidental findings. The most frequent symptom in symptomatic patients was abdominal pain in the right upper quadrant. The rarity and nonspecific symptoms of this disease underscore the difficulty in making an accurate diagnosis of HEHE.

HEHE routinely presented as multiple nodules involving both lobes of the liver with a predominantly peripheral or subcapsular growth pattern (4). Since the onset and development of HEHE were relatively insidious, most of the clinical diagnosis was at the middle or late stages. Therefore, HEHE was mostly diagnosed with multinodular or diffuse distribution, accounting for approximately 66.6%–87% of the total cases (6, 9). In this study, HEHE appeared in most cases as peripheral or subcapsular growth. Multinodular disease and the diffuse disease accounted for 72.5% of the patients, while the uninodular disease was described only in a small number of patients (27.5%).

Lung, peritoneum, lymph nodes, and bone were the most common sites of extrahepatic involvement at the time of diagnosis (6). Auxiliary examination revealed no evidence of lymph node involvement, and patients with lung metastasis at diagnosis accounted for only 9.8% patients, which was lower than the proportion reported in other literature (27%–37%) (5, 6, 10).

Hepatic capsular retraction was a morphological description of an invagination or focal flattening of the typically smooth profile of the liver capsule, which occurs in 2%–52.7% of patients (6, 11). While once believed to be exclusively correlated with hepatic malignancies, hepatic capsular retraction was in fact also associated with some benign lesions and with post-treatment changes (12). The finding of hepatic capsular retraction can help the practicing radiologist to formulate and refine a differential diagnosis in the setting of other imaging findings and clinical context. Hepatic capsule retraction was detected in eight patients in this study. However, the discovery of hepatic capsule retraction failed to help the radiologist to make an accurate diagnosis of HEHE.

HEHE usually grows around the PV, HV, and their branches. As the lesion has not invaded into the vessels, the vessels were not completely blocked yet. In CT/MRI imaging, small vessel branches can be seen within the lesion, such as right branch PV (RPV), left branch PV (LPV), right HV (RHV), and left HV (LHV), forming the so-called “tumor vessel sign” (10). This was particularly evident during the portal phase and was usually an early sign of tumor infiltration into vessels. The lollipop sign and target sign were suggestive radiological findings of HEHE. The target-like appearance of the lesion may be produced by the sclerotic fiber center, the proliferative cell layer, and the surrounding narrow vascularized zone between the lesions and the hepatic parenchyma caused by tumor infiltration and obstruction of the hepatic sinuses and small vessels (8). Contrast-enhanced MRI was the best technique for lesion characterization of the target sign, especially on T2WI and the dynamic images (13). On T2WI, a targetoid appearance consists of a core with high signal intensity, a thin ring with low signal intensity, and a peripheral halo with a slight hyperintense signal. On dynamic images, the target sign consists of a hypodense/hypointense core, surrounded by a layer of enhancement and a thin peripheral hypodense/hypointense halo. The lollipop sign was a combination of two structures: the well-defined lesion on enhanced images and the adjacent occlusive vein, as HEHE has a tendency to spread within the portal and hepatic vein branches (14). The veins should terminate smoothly at or just within the edge of the lesion, vessels that throughout the entire lesion should be excluded from the signs.

CUS was well poised to address this need due to its low cost, portability, safety, and excellent temporal resolution. The role of CUS and CEUS for liver tumor screening has been well established and supported by multiple international guidelines and thus of great significance for the early diagnosis of liver tumors (15). Similarly, CEUS can be used for the characterization of focal liver lesions in high-risk populations, and standardized criteria for CEUS have also been established. CEUS can more clearly display the blood perfusion and microvascular network distribution of organs and tumors, increase the contrast resolution of images, and dynamically and continuously evaluate the blood supply and enhancement performance of liver tumors in different enhancement stages in real time (16). CEUS can provide enhanced detection ability for multifocal HEHE, which showed a typical enhancement pattern of high enhancement in the arterial phase and low enhancement in the portal phase and delayed phase (17).

In CUS images, HEHE was similar to other liver malignancy as irregular parenchymal mass in the liver. Owing to large tumor cells and mucoid or hyaline degeneration in the interstitial tissue, the vast majority of HEHE was shown as a hypoechoic mass, as well as an isoechoic or hyperechoic mass. Most of the lesions were in the peripheral area of the right lobe of liver, some lesions could extend to the liver capsule, and most of the lesions had unclear margins. The center of the lesion may present an echoless area due to bleeding and necrosis, and calcifications may occur in approximately 20% of the patients (6).

In CEUS, most HEHE showed mild enhancement, equal to or even slightly lower than the surrounding liver parenchymal enhancement. There was significant correlation between echogenicity and tumor size. Lesions ≤ 2 cm were mostly (80.0%) uniformly enhanced as a whole, while lesions > 2 cm were mostly (91.3%) peripheral enhancement with no or low enhancement of the central part. Calcifications within the nodules were recognizable in 2 patients (20%), which was slightly higher than that reported in previous literature (6).

Contrast-enhanced CT has good temporal resolution, spatial resolution, and density resolution and has a better ability to show the blood supply characteristics of the tumor. Especially for depth lesions, it makes up for the deficiency that CEUS cannot detect blood flow signals. Diffuse tumors that meet the following criteria were very suggestive for HEHE on contrast-enhanced CT. First, lesions were large in size and slow in growth, mainly located in the periphery of the liver. Secondly, the lesion had peripheral enhancement and vascular filling, and tumors have the tendency to merge into each other. Finally, compensatory hypertrophy occurs in the unaffected liver segment, some of which may be accompanied by portal hypertension, splenomegaly, and local calcification (6).

In general, HEHE appeared as multiple nodular lesions and/or large masses on contrast-enhanced CT. Low-density pattern was the most common abnormal feature and accounted for approximately 98% of HEHE patients; the high-density and heterogeneous mixed-density lesions each accounted for only 1% of contrast-enhanced CT findings (6). In the early stage, HEHE presented as single low-density nodules, with uniform density when the lesion was ≤ 2 cm, whereas lesions > 2 cm showed peripheral or heterogeneous enhancement (10). With the progression of the disease, the single lesion may develop into multiple lesions with a tendency to coalescence. Calcification was observed in some lesions, and contrast-enhanced CT has greater sensitivity in detecting calcifications compared with CUS and CEUS. The lesions were mostly located peripheral or subcapsular of the liver. The liver capsule generally does not bulge or even shrink, forming the hepatic capsular retraction, which was a characteristic manifestation of HEHE.

Compared with contrast-enhanced CT, MRI has higher soft tissue resolution and thus has a better ability to display tumor structure more clearly. In addition, contrast-enhanced MRI can perform multi-phase dynamic scanning on patients to make qualitative diagnosis of lesions more accurate. T1WI will show a low signal while T2WI will show a high signal in tumor (18).

The characteristic contrast-enhanced MRI findings of HEHE were previously reported in the literatures, such as the tendency to coalescence, peripheral or subcapsular growth, hepatic capsular retraction, lollipop sign, and target sign. According to the results of this study, all these features were mainly seen in large lesions, except for peripheral or subcapsular growth. Moreover, although coalescent lesions were reported to be one characteristic of HEHE, about one-third of the patients were found to be nodular in this study and coalescent lesions only accounted for a small proportion of patients with multinodular or diffuse disease.

Gd-EOB-DTPA-enhanced MRI was significantly superior to contrast-enhanced CT/MRI in diagnosing small liver lesions and differentiating benign and malignant nodules (19–21). Gd-EOB-DTPA-enhanced MRI can significantly improve the sensitivity and specificity of the detection of primary liver cancer, providing an important basis for the clinical selection of appropriate personalized treatment. The application of Gd-EOB-DTPA-enhanced MRI in HEHE has been rarely studied. Xu et al. first describe the use of Gd-EOB-DTPA-enhanced MRI in the diagnosis of HEHE in a patient (22). To our knowledge, this was the first description of Gd-EOB-DTPA-enhanced MRI for diagnosing HEHE in such a large sample volume.

The technique of ^18^F-FDG PET/CT offered a significant advantage for detecting potential metastasis in HEHE patients, especially diffuse metastasis. The dual-time-point ^18^F-FDG PET/CT may be of great importance for the detection of HEHE and the determination of the severity of the disease, because some lesions not detected by early scanning can be detected by delayed scanning (23). In addition, the finding of ^18^F-FDG PET/CT was associated with tumor histopathological features and the degree of glucose uptake in HEHE tissues may be related to the size of the cells rather than the size of the tumor (24). Thus, the uptake of FDG will increase in tumors with high cell density due to increased glucose metabolism, while it will decrease in tumors with low cell density and relatively more matrix. The technique of ^18^F-FDG PET/CT has been used in a 33-year-old patient with diffuse disease, multiple low-density nodules were found, and necrotic areas could be seen in some lesions. The maximum diameter was approximately 68 mm, and the uptake of ^18^F-FDG increased, with a maximum standard uptake value (SUV) of 6.59.

It is worth mentioning that imaging studies cannot not provide a definitive diagnosis and can only lead to strong suspicion of the presence and pattern of HEHE. Positive imaging findings combined with certain features, such as more frequent young adults, more intrahepatic tumors with good clinical conditions, slow course of the disease, and calcifications in the tumor, all indicate HEHE (6). In general, it was not possible to make a specific diagnosis without biopsy in HEHE because the radiological findings were similar to those in some hepatic malignancies. The diffuse HEHE appears to have more specific diagnostic criteria, although differentiation from ICC, sHCC, MCA, and HAS may be difficult (4).

The diagnosis of HEHE was established mainly based on unique H&E staining, IHC staining, and molecular characteristics (4). HEHE mainly presents as invasive growth, consisting of epithelioid cells, dendritic cells, and intermediate cells dispersed in the hyaluronic acid-rich myxoid matrix. The HEHE cells tend to grow along vascular structures, with the potential for intravascular growth as well. The portal tracts and terminal hepatic venules remain intact despite destruction of the hepatic plates. Some lesions may be accompanied by sclerosis, necrosis, and/or calcification.

Fli-1 expressed in endothelial cells and was helpful in identifying the vascular nature of HEHE (25). CD34 was a sensitive marker of vascular tumors but not specific enough. In contrast, CD31 was a vascular tumor marker with much more specificity. Moreover, Fli-1 combined with CD31 was most helpful in distinguishing HEHE from among primary and metastatic liver tumors. All patients had at least two positive markers for vascular endothelial cells. CD31 and CD34 were positive in all the tested cases. Fli-1 was positive in 85.7% of the tested cases, while Fli-1 and CD31 were co-expressed in 12 cases.

It was important to differentiate HEHE from HAS because the latter was an aggressive tumor with poor prognosis. HAS and HEHE have similar characteristic in both H&E and IHC staining profiles. However, HEHE caused less parenchymal destruction with more sclerosis than HAS on low magnification, and, on the other hand, HAS has more nuclear pleomorphism, atypia, and mitotic activity on higher magnification (26). FVIII was another vascular endothelial cell marker and positive in almost 100% of cases, but the staining degree can be highly variable within a single lesion in different cells (1, 27). The immunoreactivity of FVIII, CD31, and CD34 was both positive in HEHE and HAS, and thus, it was difficult to distinguish HEHE from HAS by these markers above. D2-40 immunoreactivity was a useful marker of vascular lesions and was specifically expressed in HEHE among primary and metastatic liver tumors. When in conjunction with CD31, D2-40 can be critical in the confirmation of lymph vascular invasion (28, 29). D2-40 may be helpful because it was more consistently expressed in HEHE than it was in HAS (28).

It was quite difficult to differentiate HEHE from ICC and sHCC in H&E staining, since ICC typically has abundant desmoplastic stroma and gland formation or nested neoplastic cells, and sHCC also has fibrosis and eosinophilic cells with vesicular nuclei similar to that of HEHE, although those cells were usually more oncocytic. In IHC, ICC and sHCC usually have negative staining for CD31, CD34, and FVIII, even though CD34 may be positive in in cases with sinusoidal capillarization (30).

The ERG transcription factor showed a conserved expression and narrow tissue distribution in both benign and malignant vascular endothelial cells and was therefore a promising new marker in the identification of HEHE (31). CK was mainly distributed in epithelial cells and was the main skeletal protein in keratinocytes. HEHE was usually negative for CK markers with occasional exception (4). The co-expression of endothelial and epithelial markers was found in 48.8% of cases in our series. Immunoreactivity for SMA was controversial; one study did not show SMA positivity (32) while another study has demonstrated expression of SMA, mainly in EHE located in skin and soft tissues (33). Our study supported the second conclusion that SMA was confirmed positive in two out of four patients. At the same time, EMA was found positive in 8.3% of cases, which was consistent with previously published literature (34).

Nuclear expression of CAMTA1 was another useful marker to confirm a diagnosis of HEHE, with high sensitivity and specificity. This marker will likely be particularly useful for those cases in which EHE shows morphologic overlap with epithelioid angiosarcoma and epithelioid hemangioma. The nuclear expression of CAMTA1 was observed in 85% of HEH (35, 36). In the present study, nuclear CAMTA1 expression was observed in 88.5% of cases, while nuclear TFE3 expression was observed in 10.7% of cases. The CAMTA1 and TFE3 co-existed in two cases.

WWTR1-CAMTA1 gene fusion was a consistent abnormality in EHE of different anatomic sites, which was found to differentiate EHE from other morphologic mimics (37). A small subset of EHE demonstrate a YAP1 (Yes-associated protein 1)-TFE3 (transcription factor E3) fusion gene leading to overexpression of TFE3 (38). A previous study has demonstrated that WWTR1-CAMTA1 and YAP1-TFE3 can co-exist in HEH cases (39).

FISH analysis was undertaken in six patients to explore the presence of rearrangements in the previously documented 1p36 and 3q25 chromosomal regions. WWTR1-CAMTA1 fusion was proved to be positive in all the six patients (100%), which was consistent with previous literature (37, 40). No patient underwent FISH for YAP1-TFE3 fusion; thus, we were unable to assess the positive rate of the fusion gene.

Compared with other malignant liver tumors, HEHE has a good prognosis. Theoretically, LR with microscopic tumor clearance (R0) was the first choice for curative treatment of HEHE and was associated with the best prognosis (6). However, in most patients, a radical excision was impossible because of the multicentricity of the lesions. Palliative resection was not suggested because these tumors tend to behave aggressively after LR (41). LR was performed in the vast majority of patients in the present study;, none of them had palliative LR. At the last follow-up, 26 patients were still alive and 6 were DOD. The survival time ranged from 3 to 116 months.

LT was the ultimate treatment for multifocal, diffuse, unresectable, or recurrent HEHE with promotional long-term survival (42). Since most LT patients had multiple tumors or invasive growth, the 1-year and 5-year survival rates of LT patients were lower than those of LR patients (43). Although the value of LT was well established, its place in the management of HEHE was still unclear. With the aim of confirming the value of LT in the management of HEHE and to identify risk factors for post-LT recurrence, Lai et al. analyzed the outcome of transplant recipients with HEHE based on a very large patient cohort. The results confirmed the value of LT in the treatment of this rare disorder and permitted identification of patients at risk of posttransplant recurrence. Post-LT follow-up should take the HEHE-LT score into account. Extrahepatic disease localization was reconfirmed not to be a contraindication for LT. Moreover, macrovascular infiltration, a time to LT of at least 120 days, and hilar lymph node infiltration are important risk factors for recurrence (44). Only two patients underwent LT in this study, HEHE was diffuse in both patients, and no extrahepatic metastasis was found on preoperative examination. In the imaging of a 33-year-old male patient, the contrast-enhanced MRI examination revealed diffuse nodules of varying sizes with hepatomegaly, RPV invasion, and RHIVC compression. Before LT, this patient had grade C liver function and portal hypertension with massive ascites. A score of 8 was developed according to the HEHE-LT score. Unfortunately, he died of liver failure 1 month after surgery. Another 34-year-old female patient has a score of 0 at first LT. She received a second LT 66 months later since the HEHE has recurred and was still alive 103 months after the first LT.

In patients with diffuse diseases, extrahepatic metastasis, and Child–Pugh A/B grade of liver function, ST remains an alternative option with an unknown eficacy due to lack of randomized trials. ST includes chemotherapy, immunotherapy, radiotherapy, TACE, and targeted therapies (45). A handful of case reports and case series have discussed the use of ST, such as Olaratumab combined with doxorubicin (46), thalidomide (47), mTOR inhibitors after LT (48), and LR followed by interferon alpha-2 (49), and have acquired good results. Vascular endothelial growth factor (VEGF) inhibitors, such as Sorafenib (50, 51), Pazopanib (52), and Bevacizumab (53), were also used in the treatment of HEHE. For patients with extrahepatic spread, adjuvant chemotherapy may be an effective therapy to prevent recurrence after LT (54). For HEHE patients with advanced hepatic lesions who were waiting for LT, TACE may be an effective intervention. In 1989, Furui et al. reported five HEHE patients treated with TACE, four of whom survived 25–80 months and two of whom had tumor shrinkage (55). Among HEHE patients with extrahepatic metastases, those treated with TACE had a better prognosis and a lower recurrence rate than those treated with surgery approaches (56). However, clinicians should be aware of the potential adverse effects of hepatic decompensation induced by TACE, especially in cases with widespread tumor involvement and poorly preserved hepatic function. An average of three cycles were administered in our study. The initial therapeutic agents varied, and two patients had more than one regimen with secondary agents. The therapeutic regimen was changed if the patient reached the maximum tolerated dose. None of these patients had TACE. One patient had lung metastasis 2 months after the initial treatment, and another patient had lung metastasis and local progression 1 month after the initial treatment. Three patients were AWD at the last follow-up and two were DOD.

In patients with diffuse diseases, extrahepatic metastasis, and severe liver functional impairment, and in some patients with Child–Pugh A/B grade of liver function but reluctant to receive treatment, surveillance was sometimes a reasonable strategy. Patients with stable HEHE without any treatments have long-term survival or even spontaneous regression has already been reported (27). Unfortunately, because of its rarity and difficulty in diagnosis, it was impossible to accurately identify HEHE reliably in patients with non-aggressive tumors and to consider them for surveillance strategy. Twelve patients did not receive any treatment during the course of their disease. At the last follow-up, six patients were AWD and six were DOD. Four out of these six patients were DOD within 3 months, one patient was DOD 8 months later, and the last one was DOD 84 months later after diagnosis.

The prognosis of HEHE was much better than that of other hepatic malignancies. Successful LR or LT has been shown to promote long-term survival, with 5-year survival rates of 75% and 54.5%, respectively. However, the 5-year survival rates decreased sharply to 30% for patients treated with ST and 4.5% for those without treatment (6). In our study, the 5-year survival rates of LR and LT in this study were 76.0% and 50%, respectively. In patients without any treatment, the 5-year survival rate remains as high as 42.3%, which was significantly better than previously reported in the literature (6).

Chahrour et al. found the surgical approach as a favorable prognostic factor for HEHE, while age > 65 years and tumor size >10 cm were shown to be poor survival prognostic factors (57). Age group (age ≥ 60 years or 65 years), gender, MDLT, the distribution pattern of tumors (uninodular disease, multinodular disease, and diffuse disease), and the Child–Pugh grade of liver function were not associated with the outcomes of HEHE in our study.

Unlike other liver malignancies, the presence of lung metastasis was not an independent risk factor for poor prognosis. In this study, lung metastases were treated as contraindications for surgical approach, even in patients who had received LT. Even though the univariate analysis showed that the presence of lung metastasis was a poor prognostic factor, it was not independently associated with poor outcomes for HEHE.

MaVI of PV and/or HV branches was common (present in approximately 10%–40% of patients at diagnosis) during the natural history of hepatocellular carcinoma (HCC) and significantly reduces median survival when compared to patients without MVI (58). The presence of MaVI indicates that HCC has progressed into the advanced stage, and patients often miss the opportunity of LR and LT, such that only palliative treatment with a poor prognosis can be accepted (59, 60). The influence of MaVI on the treatment and prognosis of HEHE has not been fully studied. In this study, MaVI was quite common in HEHE patients, especially in the surveillance group. The presence of MaVI did not affect the physician’s treatment decision as a major factor. The univariate analysis showed that the presence of MaVI indicated a poor outcome of HEHE. However, the multivariate analysis showed that the presence of MaVI was not independently associated with poor outcomes for HEHE.

Patients in the surveillance group have experienced the worst outcome in this study, and surveillance was an independently prognostic factor for poor outcome. ST was not recommended for patients who were not candidates for surgical approaches because patients who received ST did not have better survival than those who received no treatment and just follow-up. Surgical approaches remain the first choice for patients suitable for patients with HEHE when possible.

## Conclusions

The definitive diagnosis of HEHE was dependent on histopathology, and it was not possible to make a specific diagnosis without biopsy because the radiological findings were similar to those in some hepatic malignancies. ST was not recommended for patients who were not candidates for surgical approaches, and surgical approaches should be warranted regardless of disease stage.

The retrospective nature and the small size of the data limited the generalizability of the study, designing a worldwide database that contains all data about patients with HEHE independent of their therapy, which was highly recommended.

## Data availability statement

The original contributions presented in the study are included in the article/supplementary material. Further inquiries can be directed to the corresponding author.

## Ethics statement

The studies involving human participants were reviewed and approved by Ethics Committee of West China Hospital of Sichuan University. Written informed consent for participation was not required for this study in accordance with the national legislation and the institutional requirements.

## Author contributions

LF wrote the main manuscript text as first author. ZH and ML prepared figures and tables. MX reviewed the manuscript. All authors contributed to the article and approved the submitted version.
